# Enhancing the Performance of Aqueous Solution-Processed Cu_2_ZnSn(S,Se)_4_ Photovoltaic Materials by Mn^2+^ Substitution

**DOI:** 10.3390/nano10071250

**Published:** 2020-06-28

**Authors:** Wenjie He, Yingrui Sui, Fancong Zeng, Zhanwu Wang, Fengyou Wang, Bin Yao, Lili Yang

**Affiliations:** 1 Key Laboratory of Functional Materials Physics and Chemistry of the Ministry of Education, Jilin Normal University, Siping 136000, China; jlnuhwj@163.com (W.H.); zeng740183899@163.com (F.Z.); wangzhanwu@126.com (Z.W.); wfy@jlnu.edu.cn (F.W.); llyang@jlnu.edu.cn (L.Y.); 2 State Key Laboratory of Superhard Materials and College of Physics, Jilin University, Changchun 130012, China; binyao@jlu.edu.cn

**Keywords:** Cu_2_Mn*_x_*Zn_1–*x*_Sn(S,Se)_4_, thin films, sol-gel, solar cells, photoelectric performance

## Abstract

In this work, the Cu_2_Mn*_x_*Zn_1−*x*_Sn(S,Se)_4_ (0 ≤ *x* ≤ 1) (CMZTSSe) alloy films were fabricated by a sol-gel method. Meanwhile, the effects of Mn substitution on the structural, morphological, electrical, optical, and device performance were studied systematically. The clear phase transformation from Cu_2_ZnSn(S,Se)_4_ (CZTSSe) with kesterite structure to Cu_2_MnSn(S,Se)_4_ (CMTSSe) with stannite structure was observed as *x* = 0.4. The scanning electron microscope (SEM) results show that the Mn can facilitate the grain growth of CMZTSSe alloy films. Since the *x* was 0.1, the uniform, compact, and smooth film was obtained. The results show that the band gap of the CMZTSSe film with a kesterite structure was incessantly increased in a scope of 1.024–1.054 eV with the increase of *x* from 0 to 0.3, and the band gap of the CMZTSSe film with stannite structure was incessantly decreased in a scope of 1.047–1.013 eV with the increase of *x* from 0.4 to 1. Meanwhile, compared to the power conversion efficiency (PCE) of pure CZTSSe device, the PCE of CMZTSSe (*x* = 0.1) device is improved from 3.61% to 4.90%, and about a maximum enhanced the open-circuit voltage (*V_OC_*) of 30 mV is achieved. The improvement is concerned with the enhancement of the grain size and decrease of the Cu instead of Zn (Cu_Zn_) anti-site defects. Therefore, it is believed that the adjunction of a small amount of Mn may be an appropriate approach to improve the PCE of CZTSSe solar cells.

## 1. Introduction

Cu_2_ZnSn(S,Se)_4_ (CZTSSe) has drawn great attention due to tunable band gaps and high absorption coefficient (>10^4^ cm^−1^) [1,2,3]. Recently, the highest power conversion efficiency (PCE) of CZTSSe device is 12.6% [4,5], which is significantly lower than the theoretical prediction value and the PCE of Cu(In,Ga)Se_2_ (CIGS) device [6]. Compared with the word record CIGS devices, the open-circuit voltage (*V_OC_*) deficit has been considered as a primarily restrictive factor of PCE in CZTSSe solar cells [7]. Although the cause for the *V_OC_* deficit in CZTSSe solar cells is still an open question, the random distribution of Cu/Zn atoms at 2c and 2d positions is considered one of the significant reasons for low *V_OC_* [8]. It is reported that band tailing states are induced by a great quantity of the Cu instead of Zn (Cu_Zn_) defects near the band edge, and, thereby, restrict *V_OC_* [9]. Therefore, eliminating Cu_Zn_ anti-site defects is deemed a key challenge to break the *V_OC_* bottleneck [10].

Recently, the cation substitution has attracted people’s interest as a method to lessen Cu_Zn_ anti-site defects of CZTSSe film. The replacement of Cd for Zn can significantly increase the PCE of CZTSSe devices from 5.3% to 9.24%, which indicates that cation replacement of Zn has great potential for improving the PCE of CZTSSe solar cells [11,12]. Replacing Cu with Ag in CZTSSe is also an efficacious method to decrease the concentration of anti-site defects, which, thereby, improves device performance [13,14,15]. However, the general substitution elements such as Ag and Cd are mostly rare or toxic, which deviates from the profit of CZTSSe. In addition, in some works, cation substitution has been proven to be beneficial to improve the crystallinity and increase the carrier concentration of CZTSSe thin film [16,17,18]. However, due to the mismatch of ion radius and high formation energy for Cd and Ag, these doping elements have difficulty in entering the crystal lattice and can only exist on the surface of grain boundaries of CZTSSe [19]. According to these issues, the doping of Cd and Ag elements is not the most appropriate, so it is still necessary to establish a suitable cation substitute to decrease cation anti-site defects and improve properties of the CZTSSe absorber layer.

It is also possible to replace Zn with ideal substitutes such as Fe, Co, Ni, and Mn [20]. These cations are more environmentally-friendly and richer than previous alternatives. These candidate cations should be alloyed from pure-Zn to pure-substitute cations. The ion radius of Mn^2+^ is closer to that of Zn^2+^ when compared with other similar atoms such as Fe, Co, and Ni, so Mn^2+^ can substitute the Zn^2+^ site in CZTSSe crystal lattices instead of being isolated on the CZTSSe surfaces and crystal boundaries. Furthermore, the reserves of Mn in the crust of earth are not only substantial, but environmentally-friendlier than the toxic Cd. Therefore, Mn element doping is a simple and effective way to implement the optimization of the CZTSSe film. However, the synthesis of the Cu_2_Mn*_x_*Zn_1−*x*_Sn(S,Se)_4_ (0 ≤ *x* ≤ 1) (CMZTSSe) films and the influence of Mn doping on properties of CMZTSSe thin films are rarely reported so far. Therefore, it is necessary to systematically study the performance of the CMZTSSe alloyed film, which will offer a solid foundation to obtain the solar cells with the higher PCE. In the present work, we first prepared CMZTSSe thin films with different Mn concentrations by the sol-gel method, and systematically researched the performance of CMZTSSe alloy films in detail. The performance of CMZTSSe solar cells has also been systematically studied. Lastly, CMZTSSe solar cells of close to 5% efficiency was achieved with *x* = 0.1, which is characterized by about 30 mV improvement in *V_OC_*.

## 2. Experiments

### 2.1. CMZTSSe Thin Film Preparation

The CMZTSSe thin films were made by using a two-step method, sol-gel, and post selenization. First, C_4_H_6_CuO_4_∙H_2_O (0.27 mol·L^−1^), ZnCl_2_, and MnCl_2_ (0.18 mol·L^−1^), SnCl_2_·2H_2_O (0.15 mol·L^−1^), and CH_4_N_2_S (1.2 mol·L^−1^) were dissolved in dimethyl sulfoxide (10 mL) to form aqueous precursor solution at room temperature. Mn content in precursor solution was controlled by the Mn/(Zn + Mn) ratio. The molar ratio of Mn to (Zn+Mn) in solution varies from *x* = 0, 0.1, 0.2, 0.3, 0.4, 0.6, 0.8, and 1. Cu_2_Mn*_x_*Zn_1−*x*_SnS_4_ (CMZTS) precursor solution was spin-coated on soda-lime glass (SLG) at 3000 rpm for 30 s and preheating for 5 min at 300 °C. Subsequently, in order to yield the CMZTSSe thin films, the CMZTS precursor films and 0.2 g selenium powder were loaded in the graphite susceptor and then annealed in the rapid heat treatment furnace at 540 °C under N_2_ flow for 900 s.

### 2.2. Solar Cell Fabrication

The solar cells were fabricated by the routine process. The device with the structure of Mo/CMZTSSe/CdS/ZnO/ITO/Ag was prepared. Therefore, the Mo layer (600 nm) was sputtered as a back contact layer, and the CMZTSSe absorber layer of 1.0 μm is fabricated on the Mo substrate by the sol-gel method. Subsequently, a 60-nm thick CdS film was fabricated by chemical bath deposition (CBD) on the CMZTSSe absorber. After that, a 50-nm thick ZnO and a 250-nm thick indium tin oxide (ITO) were fabricated on CdS buffer layers by radio frequency (RF) magnetron sputtering. Lastly, the Ag electrode was deposited by thermal evaporation. The solar cell is formed by isolation with mechanical scribing. The total area of each solar cell is 0.19 cm^2^.

### 2.3. Thin Film and Device Characterization

The X-ray diffraction (XRD) (XRD, Rigaku Corporation, Tokyo, Japan) was conducted to measure the crystal structure of CMZTSSe film. Raman spectra were recorded by Renishaw system (Renishaw, London, UK) using a 514-nm excitation wavelength. The chemical composition of CMZTSSe thin film was detected by X-ray Photoelectron Spectroscopy (XPS) (XPS, Thermo Fisher Scientific, Waltham, MA, USA) with an Al Kα X-ray source. Field emission scanning electron microscope (FE-SEM) images were obtained by Hitachi S-4800, and the energy dispersive spectroscopy (EDS) analyzer was collected in FE-SEM (Hitachi S-4800, JEOL Ltd., Tokyo, Japan). The UV-vis-near-infrared spectrophotometer (UV-3101PC, Tokyo, Japan) was used to analyze the optical properties of the films. The electrical performance was tested using the Hall-effect system with Van der Pauw configuration. The curves of the photocurrent and the voltage (J-V) were measured under AM 1.5 G simulated sunlight illumination (Model 91160, Newport, Irvine, American).

## 3. Results and Discussion

**Figure 1:**
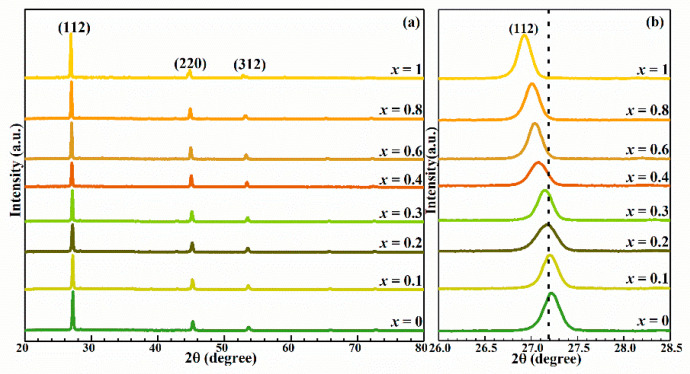
(**a**) XRD spectra of Cu_2_Mn*_x_*Zn_1−*x*_Sn(S,Se)_4_ (0 ≤ *x* ≤ 1) thin films. (**b**) Enlarged view of the corresponding (112) diffraction peaks of the Cu_2_Mn*_x_*Zn_1−*x*_Sn(S,Se)_4_ (0 ≤ *x* ≤ 1) thin films.

Figure 1a showed the XRD diffraction patterns of CMZTSSe (0 ≤ *x* ≤1) thin films. The CMZTSSe film shows characteristic diffraction peaks at 28.53°, 47.33°, and 56.17°, corresponding to the (112), (220), and (312) planes of Cu_2_ZnSnS_4_ (CZTS) with a tetragonal kesterite structure [21,22]. No secondary phases of Zn-, Sn-, Cu-, and Mn-based selenization compounds were observed in all CMZTSSe films, which indicated Mn does not exist as impurity atoms in the film. In other words, Mn^2+^ will be evenly substituted to the Zn^2+^ position or interstitial position in the CZTSSe lattice. In addition, Figure 1b displayed the enlarged (112) diffraction peaks. The (112) diffraction peaks moved from 28.53° to 26.92° with the increase of the *x* value because there is an inverse relationship between diffraction angle *θ* and crystal lattice parameter, which indicates that the crystal lattice parameter of the CMZTSSe increases. According to the International Center for Diffraction Data standards of JCPDS#26-0542, the (112) diffraction peak of pure Cu_2_MnSn(S,Se)_4_ (CMTSSe) with stannite structure was located at 26.92°. When the *x* increased to 0.4, it was observed that the (112) peak located around 27.23° is much closer to the (112) peak located around 26.92° from the pure CMTSSe, which suggests that the structure of CMZTSSe (*x* = 0.4) may be changed from the kesterite structure of CZTSSe to the stannite structure of CMTSSe. Since *x* is further increased, it was found that the diffraction angle of (112) peak continues to move to a smaller angle. We suspect that the CMZTSSe (*x* = 0.4, 0.6, 0.8) films all have the stannite structure of CMTSSe. When *x* = 1, the CMZTSSe film entirely consists of the CMTSSe phase with the stannite structure. As we all know, there is a strong correlation between the change of lattice parameters and the size of ions [16]. Therefore, we speculate the cause for the increase in the lattice parameters of CMZTSSe is that the covalent radius of Mn^2+^ (0.80 Å) is greater than that of Zn^2+^ (0.74 Å), and Mn^2+^ substituted Zn^2+^ position in the CZTSSe lattice.

**Figure 2:**
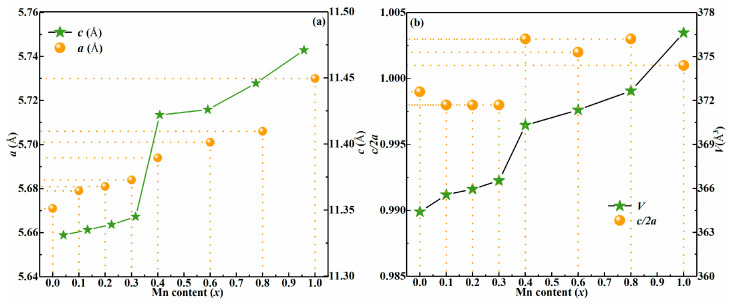
(**a**) The variation of a-axis lattice constant (*a*) and c-axis lattice constant (*c*) in CMZTSSe (0 ≤ *x* ≤ 1) alloy films. (**b**) The evolution of the unit cell volume (*V*) and the lattice parameters (*η* = *c*/2*a*) with the Mn content for the Cu_2_Mn*_x_*Zn_1−*x*_Sn(S,Se)_4_ (0 ≤ *x* ≤ 1) thin films.

To study the impact of Mn substitution on crystal structures, the XRD results of CMZTSSe films were further analyzed. The lattice parameters of tetragonal structures can be extracted by using the relation below.

$$\frac{1}{{d_{\mathrm{hkl}}}^{2}}=\frac{h^{2}{+k}^{2}}{a^{2}}+\frac{l^{2}}{c^{2}}$$

where *d*_hkl_ is the plane distance between (hkl) planes [23]. Figure 2a displays an a-axis lattice constant (*a*), and c-axis lattice constant (*c*) in CMZTSSe (0 ≤ *x* ≤ 1) alloy films. It was found that the lattice constant *a* gradually increases from 5.671 to 5.730 Å while increasing *x* from 0 to 1. Meanwhile, the lattice constant *c* also increases from 11.331 Å to 11.471 Å. The increase in the lattice constants is mainly related to the ionic radius size. The ionic size of the Mn^2+^ cation is about 0.80 Å whereas it is 0.74 Å for Zn^2+^ in CZTSSe film. Therefore, it can be deduced that Mn mainly occupies Zn sites for CMZTSSe film. This aligns with the XRD results. Figure 2b shows the unit cell volume (*V*) and lattice parameters (*η* = *c*/2*a*) in CMZTSSe (0 ≤ *x* ≤ 1) alloy films, respectively. As shown in Figure 2b, the unit cell volume of CMZTSSe film increases with the increase of *x* from 0 to 1, which is due to the substitution of larger Mn instead of the smaller size Zn in the crystal lattice [18]. In Figure 2b, it was found that the *η* = *c*/2*a* change as the Mn content increases. Quaternary chalcogenide semiconductor structure studies have shown that the *η* has a different value with symmetry, i.e., *η* < 1 for kesterite structure and *η* > 1 for stannite structure of CZTSSe [24,25]. On the basis of Figure 2b, it was discovered that *η* values of CMZTSSe (0 ≤ *x* ≤ 0.3) films are all less than 1. Consequently, it is testified that CMZTSSe have the kesterite structure at 0 ≤ *x* ≤ 0.3. However, we also discovered that *η* = *c*/2*a* values are more than 1 for all the CMZTSSe (0.4 ≤ *x* ≤ 1) films. Consequently, it is proven that CMZTSSe thin films have the stannite structure at 0.4 ≤ *x* ≤ 1. This also confirms the inference from the above XRD results about the phase transition at *x* = 0.4.

**Figure 3:**
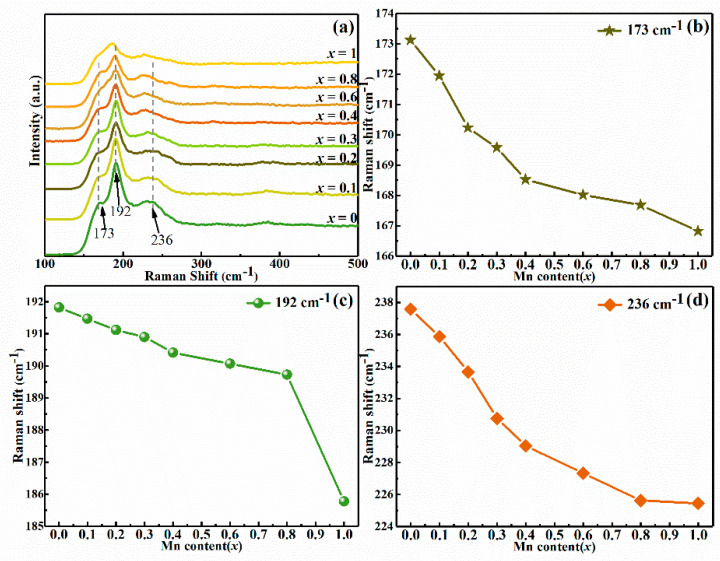
(**a**) Raman spectra of the Cu_2_Mn*_x_*Zn_1−*x*_Sn(S,Se)_4_ (0 ≤ *x* ≤ 1) thin films. The variations of the Raman peaks position with the Mn content for the Cu_2_Mn*_x_*Zn_1−*x*_Sn(S,Se)_4_ (0 ≤ *x* ≤ 1) alloy thin films: (**b**) 173 cm^−1^, (**c**) 192 cm^−1^, and (**d**) 236 cm^−1^.

For the sake of detecting a probable secondary phase in CMZTSSe films, the Raman spectra of CMZTSSe alloy films with different Mn content were surveyed and shown in Figure 3. When *x* = 0, the Raman peaks situated at 173, 192, and 236 cm^−1^ are clearly found and the peaks at 173, 192, and 236 cm^−1^ are part of the A_1_ vibrational mode from the CZTSSe phase with a kesterite structure, which is consistent with the literature reported previously [26]. We also noticed that no possible impurity peaks appeared, which implies that a pure CZTSSe film was prepared. When *x* = 1, the Raman peaks located at 167, 185, and 225 cm^−1^ were clearly observed for the pure CMTSSe phase with a stannite structure. The position of Raman peaks is some deviation compared to the Raman peaks (173, 188, and 234 cm^−1^) from A_1_ vibrational mode for the CMTSSe phase reported in previous literature [26]. This may be due to the difference of Se content in film, which is caused by the selenization process. To survey the Mn influence on Raman spectra, the variation of Raman peak position of CMZTSSe alloy thin film with the Mn content is shown in Figure 3b–d. All Raman peaks of CMZTSSe films shift toward the low frequency direction with increasing Mn content. This phenomenon can be interpreted by incorporating Mn into the CZTSSe crystal lattice, which leads to the changes in atomic vibrations and reflects the influence of the replacement Zn^2+^ with heavier and bigger Mn^2+^.

**Figure 4:**
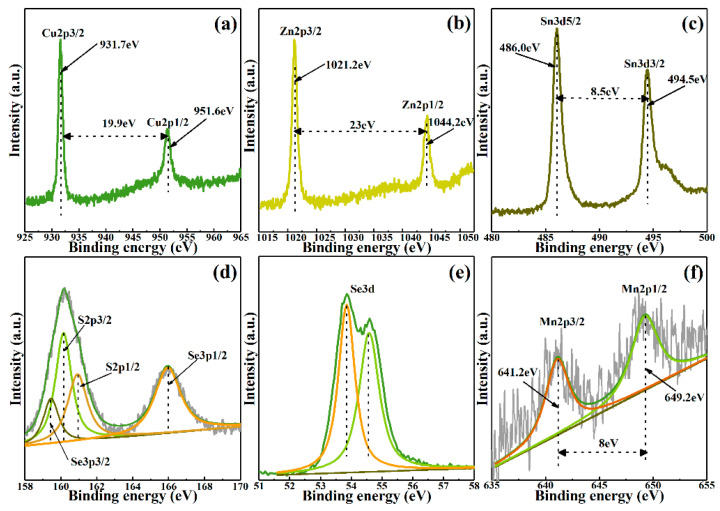
X-ray Photoelectron Spectroscopy (XPS) spectrum of Cu_2_Mn*_x_*Zn_1−*x*_Sn(S,Se)_4_ (*x* = 0.1) thin films: (**a**) Cu, (**b**) Zn, (**c**) Sn, (**d**) S, (**e**) Se, and (**f**) Mn.

The X-ray Photoelectron Spectroscopy (XPS) measurement was used to distinguish the effect of Mn doping on chemical composition in CMZTSSe alloy films. The high resolution scanning XPS spectra of representative CMZTSSe (*x* = 0.1) thin film for Cu 2p, Zn 2p, Sn 3d, S 2p, Se 3d, and Mn 2p are shown in Figure 4a–f. Figure 4a showed that two peaks of Cu 2p_3/2_ and Cu 2p_1/2_ at 931.7 eV and 951.6 eV, respectively, and the peak separation value is 19.9 eV, which conform well to the reported values for Cu^+^. Furthermore, the binding energy of Cu^2+^ is usually located at 942 eV and is shown as a typical satellite peak, which is not observed in the current XPS spectrum. Therefore, the valence value of Cu in the prepared film is +1 (Cu^+^) [27]. At the same time, two peaks at 1021.2 and 1044.2 eV are attributed to Zn 2p_3/2_ and Zn 2p_1/2_, as depicted in Figure 4b. In addition, the splitting value is 23 eV and the results show that the Zn is oxidized to Zn^2+^ [28]. It can be clearly found that two peaks of Sn 3d_5/2_ and Sn 3d_3/2_ at 486.0 eV and 494.5 eV are shown in Figure 4c, respectively, and the splitting value is 8.5 eV. The result conforms to segregation value of Sn^4+^ [29]. This means that Sn^2+^ is oxidized to Sn^4+^ and Cu^2+^ is reduced to Cu^+^ during the film growth process [30]. The XPS spectra of S 2p for CMZTSSe alloy films are shown in Figure 4d. As a result of the S 2p core level overlaps with Se 3p, the four peaks of 159.4, 160.1, 161.0, and 166.0 eV were obtained by Gaussian fitting means, which belongs to Se 2p_3/2_, S 2p_3/2_, S 2p_1/2_, and Se 2p_1/2_, respectively. Two peaks concentrated on 160.1 eV and 161.0 eV, corresponding to S 2p_3/2_ and S 2p_1/2_, which conforms to 160–164 eV range of S in sulfide phases [31]. It can be seen from Figure 4e that the two different sub-spectra with binding energy of 53.8 eV and 54.6 eV were obtained by fitting the XPS spectrum of Se 3d, which was ascribed to Se 3d_3/2_ and Se 3d_1/2_, respectively [32]. The binding energy of the Se 3d well conforms to the reported values for Se^2−^. As shown in Figure 4f, two peaks are concentrated on 641.2 eV and 649.2 eV, respectively, which is attributed to Mn 2p_3/2_ and Mn 2p_1/2_ [33]. The splitting value is 8 eV, which is conformed to the splitting value of Mn^2+^. This shows that Mn has been successfully incorporated into CZTSSe, occupying the position of Zn atoms, and, thereby, forming a CMZTSSe alloy film.

We measured the CMZTSSe (0 ≤ *x* ≤ 1) alloy thin films with different Mn content by energy dispersive spectroscopy (EDS), and obtained the chemical composition of the film and shown in Table 1. The measurement results show that the Mn content increased while Zn decreases significantly with the *x* increasing. The Zn content decreases with Mn content increasing in CMZTSSe films, which conforms to the conclusion obtained by XRD and XPS in which Zn atoms are replaced by Mn atoms. Furthermore, it can also be found from Table 1 that, when compared with pure CZTSSe film, the Cu content increased and the Sn content reduced for the CMZTSSe thin films (*x* = 0.1), which resulted in an increase in the ratios of Cu/(Zn + Mn + Sn) and (Zn + Mn)/Sn. The addition of the Cu/(Zn + Mn + Sn) ratio may lead to a reduction of Cu_Zn_ anti-site defect and the increase of Cu_Zn_ + Sn_Zn_ deep donor defect. This resulted in the decrease of the carrier concentration [34,35]. In the current work, it is found that the carrier concentration decreases with increasing Mn doping content, which can be confirmed in the later Hall measurement results.

**Figure 5:**
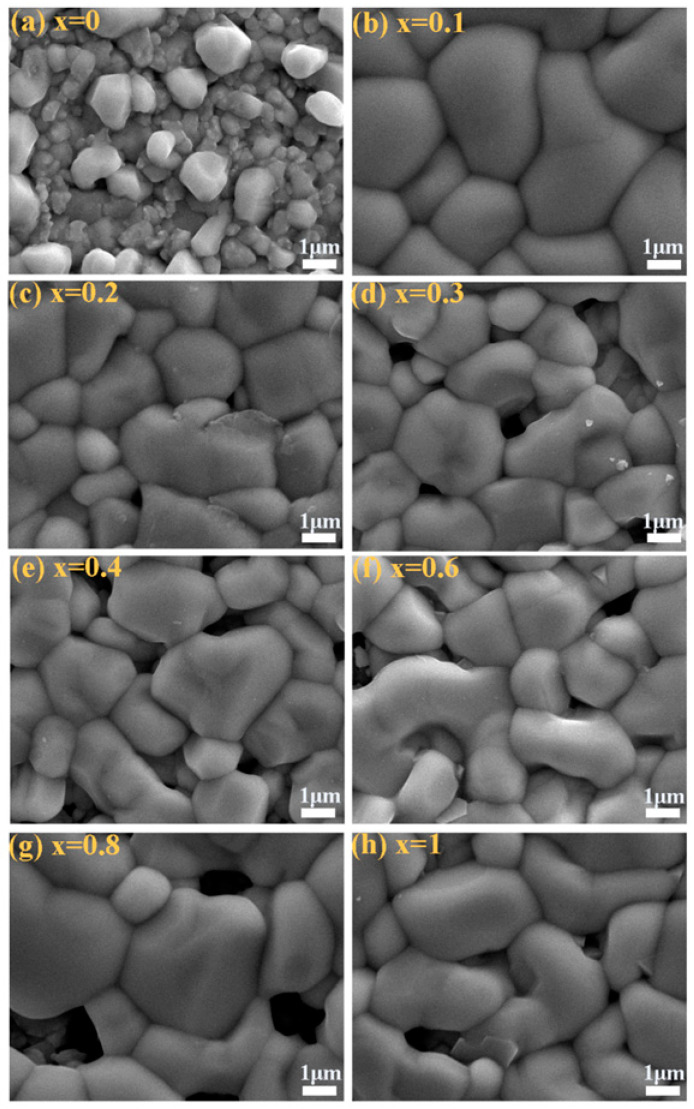
SEM images of the Cu_2_Mn*_x_*Zn_1−*x*_Sn(S,Se)_4_ (0 ≤ *x* ≤ 1) alloy thin films: (**a**) *x* = 0, (**b**) *x* = 0.1, (**c**) *x* = 0.2, (**d**) *x* = 0.3, (**e**) *x* = 0.4, (**f**) *x* = 0.6, (**g**) *x* = 0.8, (**h**) *x* = 1.

Figure 5a–h display the SEM images of CMZTSSe (0 ≤ *x* ≤ 1) alloy films. We describe the significant changes in the grain size upon Mn-substitution. It was found that the grain size increased significantly when Mn was doped into the film. It can be clearly seen that the surface morphology of CZTSSe film is considerably rough, as shown in Figure 5a. The CMZTSSe (*x* = 0.1) film surface presents a very flat and compact state, which is good for improving the PCE of solar cells, as shown in Figure 5b. When *x* = 0.2, the surface morphology of the film becomes slightly rough, as shown in Figure 5c. In addition, Figure 5d shows the holes appear on the film surface at *x* = 0.3. More and more holes are observed when Mn content increases. The grain size of CMTSSe film is basically the same as that of the film with high Mn content, but there is a certain void observed from Figure 5h. According to the above analysis, it was found that the suitable introduction of Mn^2+^ is beneficial to the grain growth. Especially at *x* = 0.1, the CMZTSSe film has not only the largest grain size, but also smooth and compact surface morphology.

**Figure 6:**
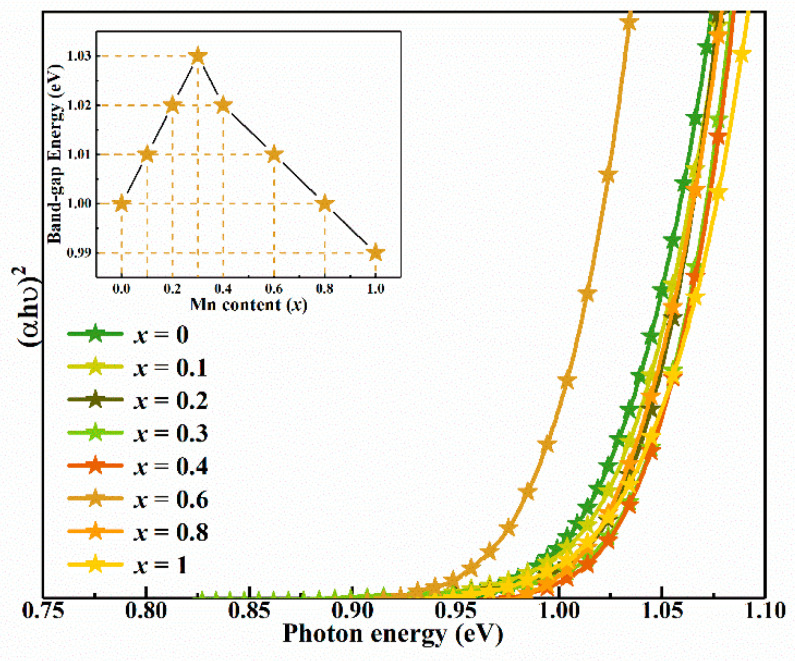
Plot of (*αhυ*)^2^ against *hυ* for the Cu_2_Mn*_x_*Zn_1−*x*_Sn(S,Se)_4_ (0 ≤ *x* ≤ 1) alloy thin films. Inset: Band gap variation as a function of the Mn content.

To analyze the influence of Mn content on band gap (*E_g_*) of CMZTSSe alloy films, the absorption spectra were obtained by the UV-vis-near-infrared (UV-vis-NIR) spectrophotometer. Figure 6 shows the variation of (*αhν*)^2^ versus *hν* for the CMZTSSe film with different Mn contents. According to the band theory of solids, the *E_g_* value of the semiconductor thin films can be calculated from the optical absorption spectra by using Tauc’s relation.

*αhυ* = *A*(*hυ* − *E_g_*)*^n^* (1)

The coefficient *A* is a constant, and *n* assumes the values 1/2, 2, 3/2, and 3 for allowed direct, allowed indirect, forbidden direct, and forbidden indirect transitions, respectively [36]. The CMZTSSe is considered more suitable for direct band gap energy material. Hence, *n* = 1/2 is employed for this work, and the relationship between the absorption coefficient (*α*) and band gap can be described by the following equation.

*αhυ* = *A*(*hυ* − *E_g_*)^1/2^ (2)

By utilizing the data in Figure 6 and Equation (2), the *E_g_* can be calculated by extrapolating the linear portion of the curves to intercept the energy x-axis. When (*αhυ*)^2^ = 0, *E_g_* = *hυ*. As shown in the inset of Figure 6, the determined *E_g_* values of CMZTSSe films are 1.024, 1.030, 1.037, 1.054, 1.047, 1.042, 1.033, and 1.013 eV at *x* = 0, 0.1, 0.2, 0.3, 0.4, 0.6, 0.8, and 1, respectively. The *E_g_* values of CMZTSSe alloyed thin films gradually increases with 0 ≤ *x* ≤ 0.3. However, the band gap values decrease gradually with 0.4 ≤ *x* ≤ 1. This likely has a bearing on variations in the electronic structure of the system. *E_g_* of CZTSSe semiconductor is mostly determined by the conduction band minimum (*CBM*) and valence band maximum (VBM) (i.e., CBM = VBM + *E_g_*). In CZTS and CZTSe, according to the first-principles calculation, the VBM of CZTS (CZTSe) is mostly influenced by the antibonding of S 3p (Se 4p) and Cu 3d orbitals [37]. While the CBM of CZTS (CZTSe) is mostly correlated to the antibonding of S 3p (Se 4p) and Sn 3d orbitals. At the same time, in Cu_2_MnSnS_4_ (CMTS) and Cu_2_MnSnSe_4_ (CMTSe), the VBM of CMTS (CMTSe) is mostly influenced by the antibonding of S 3p (Se 4p) and Cu 3d (Mn 5d) orbitals. The CBM of CMTS (CMTSe) is mostly correlated to the antibonding of S 3p (Se 4p) and Mn 5d orbitals [38]. Therefore, the structure of CMZTSSe film is a kesterite structure when the *x* value increased from 0 to 0.3, as a small amount of Mn was doped into CZTSSe film. A small amount of Mn may not hybridize with S/Se to substitute Sn as the main cation, which is the answer for the formation of the CBM. Therefore, increase of the band gap may be attributed to the low Mn content in the film, which weakens the hybridization between Sn and S/Se in the conduction band. However, the band gap value gradually decreases with 0.4 ≤ *x* ≤ 1. This may be due to the *E_g_* of CMTSSe being smaller than the *E_g_* of CZTSSe and the structure of the film changing from kesterite to a stannite phase when the *x* value was increased from 0.4 to 1. A large amount of Mn was doped into CZTSSe film. The Mn with a larger cation radius will hybridize with S/Se, and the hybridization is very strong, which results in the reduction of *E_g_*.

Table 2 shows the electrical properties of CMZTSSe films measured by Hall effect measurements at room temperature. We repeatedly measure the same sample to ensure the accuracy of the electrical performance of the film. On the basis of analysis results of structure, the CMZTSSe (0 ≤ *x* ≤ 1) film has kesterite structure when the range of *x* is between 0 and 0.3. Therefore, the electrical properties of CMZTSSe alloy films (0 ≤ *x* ≤ 0.3) are studied. The CZTSSe film exhibited the p-type (hole mediated) conductivity, as shown in Table 2. According to some reports, the Cu replaces the Zn site (Cu_zn_), which is the major acceptor in CZTSSe thin film, making it exhibit p-type conductivity. Compared with the pure CZTSSe films, the carrier concentration of CMZTSSe films decreased slightly and the hall mobility increased when *x* = 0.1. It can be concluded from the results of XRD, Raman, and EDS that Mn has been doped into the CZTSSe and the Sn content decreased, which resulted in Cu/(Zn + Mn + Sn) and (Zn + Mn)/Sn ratios increasing in the CMZTSSe thin films when *x* = 0.1, which lead to the decrease of Cu_Zn_ anti-site defects. Therefore, the increase of the hall mobility is attributed to the hole concentration of Cu_Zn_ decreases with the reduction of Cu_Zn_ anti-site defects. As the *x* increases from 0.1 to 0.3, all CMZTSSe films are p-type conductivity, but the hall mobility of CMZTSSe films clearly reduced. This may be ascribed to the deterioration of the crystallization quality of the CMZTSSe film with the increase of Mn content, as shown in the SEM results.

**Figure 7:**
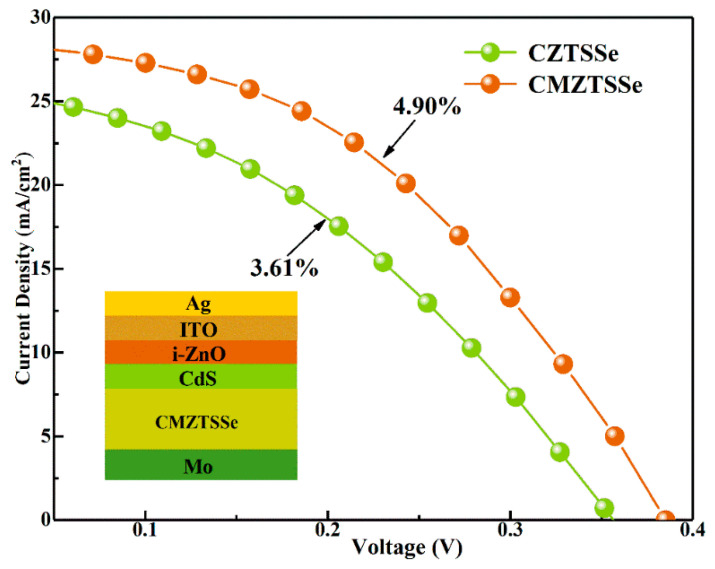
Current-voltage characteristics of the CZTSSe and Cu_2_Mn*_x_*Zn_1−*x*_Sn(S,Se)_4_ (*x* = 0.1) devices. Inset: The device schematic structure of Cu_2_Mn*_x_*Zn_1−*x*_Sn(S,Se)_4_ (*x* = 0.1).

According to the experimental results above, the CMZTSSe (*x* = 0.1) film has not only the largest grain size, but also smooth and compact surface morphology, meanwhile it also has a higher mobility and a lower Cu_Zn_ anti-site defects content, which is appropriate as the absorption layer of CZTSSe devices. In order to study the influence of Mn substitution, the CZTSSe and CMZTSSe (*x* = 0.1) solar cells are prepared under the same experimental conditions and the device performance are shown in Figure 7. The inset of Figure 7 displays a schematic structural diagram of the CMZTSSe (*x* = 0.1) device with the SLG/Mo/CMZTSSe/CdS/ZnO/ITO/Ag structure. Table 3 shows the corresponding device parameters. It is clear that the CMZTSSe (*x* = 0.1) solar cell achieves a PCE of 4.90% with the open-circuit voltage (*V_OC_*), short-circuit current density (*J_SC_*), and fill factor (*FF*) values of 385 mV, 28.64mA/cm^2^, and 44.40%, respectively. The *J_SC_* and *FF* values of the CMZTSSe (*x* = 0.1) device are 11.1% and 12.8% higher than pure CZTSSe solar cell. After calculation, the *R_S_* and *R_Sh_* of CMZTSSe device are 1.5 Ω∙cm^2^ and 366.3 Ω∙cm^2^, respectively, while the *R_s_* and *R_Sh_* of CZTSSe solar cells are 2.9 Ω∙cm^2^ and 353.1 Ω∙cm^2^, respectively. The reduction of *R_S_* and the improvement of *R_Sh_* for the CMZTSSe device are mostly due to the increase of grain size and the decrease of defect density. The decrease in *R_S_* and improved *R_Sh_* result in the improvement of *J_SC_* and *FF* [14]. The *V_OC_* of CMZTSSe device increases from 356 to 385 mV in comparison with the CZTSSe device. According to the EDS results, this may be due to the decrease of the Cu_Zn_ anti-site defect states the concentration in CMZTSSe film, which decreases the band-tailing and produce larger band bending at the interface of the absorber and buffer layer. This results in the improvement of *V_OC_* and improvement of the device performance. Thus, Mn substitution improves the *V_OC_* of the CZTSSe device, which can be used as a solution to enhance the integral performance of thin film solar cells. In addition, the CMZTSSe (*x* = 0.1) solar cells can achieve the higher *J_SC_*, which is mainly related to the smaller *E_g_* of CMZTSSe thin films. When the *E_g_* of the absorption layer is small, the spectral response range of the device is larger, which leads to a larger current density. The Jsc obtained in this work is comparable to that reported in other literatures about the CZTSSe film solar cells with high efficiency [39,40]. This also further improves that doping a small amount of Mn is an appropriate way to improve the efficiency of CZTSSe solar cells.

## 4. Conclusions

We reported the synthesis of CMZTSSe (0 ≤ *x* ≤ 1) alloy films by the sol-gel method. The results show that the substitution of Mn significantly improves the crystal growth of CZTSSe thin films. In CZTSSe thin films, the replacement of Zn with Mn is exhibited to cause structural transformation at *x* = 0.4, which is mostly ascribed to divergence of the atomic radius. At the same time, the change of Mn substitution content will also affect the band gap of CMZTSSe thin film. The CMZTSSe thin films have an average size that increases from 0.85 to 2.35 μm as *x* increases from 0 to 0.8. It was found that the suitable doping of Mn^2+^ is beneficial to the growth of grains. The grain size of the CMZTSSe (*x* = 0.1) film has largest grain size, and the surface morphology is smooth and dense. As expected, replacement of Zn with Mn can suppress the production of Cu_Zn_ anti-site defects, which leads to improvements of the *V_OC_*. The reduced *R_S_* and improved *R_Sh_* result in the improvement of *J_SC_* and *FF*. The change trends of *J_SC_*, *FF*, and *V_OC_* results in *PCE* improving from 3.61% to 4.90% for the CMZTSSe device with the *V_OC_*, *J_SC_*, and *FF* values of 385 mV, 28.64 mA/cm^2^, and 44.40%, respectively. It is concluded that replacement of Mn is a promising method to alleviate the *V_OC_* deficit and increase performance of CZTSSe photovoltaic devices.

## Figures and Tables

**Table 1:** Summary of the EDS results of Cu_2_Mn*_x_*Zn_1−*x*_Sn(S,Se)_4_ (0 ≤ *x* ≤ 1) thin films with various Mn contents.

Sample	Cu (% at)	Mn (% at)	Zn (% at)	Sn (% at)	S (% at)	Se (% at)	Cu/(Zn + Mn + Sn)	(Mn + Zn)/Sn	Mn/(Mn + Zn)
*x* = 0	23.49	0	15.64	11.48	2.88	46.51	0.87	1.36	0
*x* = 0.1	24.01	1.30	14.79	11.32	2.42	46.16	0.88	1.42	0.08
*x* = 0.2	24.23	2.22	13.36	11.53	2.63	46.03	0.89	1.35	0.14
*x* = 0.3	24.34	3.26	11.67	11.53	2.66	46.56	0.91	1.29	0.22
*x* = 0.4	24.24	4.64	10.38	11.59	3.72	45.43	0.91	1.29	0.31
*x* = 0.6	25.97	6.23	7.61	11.18	3.72	45.28	1.04	1.23	0.45
*x* = 0.8	26.38	9.33	3.82	11.14	3.02	46.32	1.08	1.18	0.71
*x* = 1	27.50	12.34	0	11.31	2.90	45.98	1.16	1.09	1

**Table 2:** Electrical properties of the Cu_2_Mn*_x_*Zn_1−*x*_Sn(S,Se)_4_ (0 ≤ *x* ≤ 0.3) thin films measured by Hall effect measurements.

Samples	Resistivity (Ω·cm)	Carrier Concentration (cm^−3^)	Mobility (cm^2^∙V^−1^∙s^−1^)	Type
*x* = 0	5.3 × 10^1^	4.2 × 10^16^	2.8	p
*x* = 0.1	8.7 × 10^1^	2.3 × 10^16^	3.2	p
*x* = 0.2	8.6 × 10^1^	4.6 × 10^16^	1.6	p
*x* = 0.3	6.4 × 10^1^	6.3 × 10^16^	1.5	p

**Table 3:** Parameters of the device performance.

Device	Active Area	*V_OC_* (mV)	*J_SC_* (mA/cm^2^)	*FF* (%)	*PCE* (%)	*R_s_* (Ω∙cm^2^)	*R_sh_* (Ω∙cm^2^)
CZTSSe	0.19 cm^2^	356	25.77	39.35	3.61	2.9	353.1
CMZTSSe (*x* = 0.1)	0.19 cm^2^	385	28.64	44.40	4.90	1.5	366.3
